# Control of Lyme borreliosis and other *Ixodes ricinus*-borne diseases

**DOI:** 10.1186/s13071-018-2744-5

**Published:** 2018-03-06

**Authors:** Hein Sprong, Tal Azagi, Dieuwertje Hoornstra, Ard M. Nijhof, Sarah Knorr, M. Ewoud Baarsma, Joppe W. Hovius

**Affiliations:** 1 Centre for Zoonoses & Environmental Microbiology, Centre for Infectious Disease Control, National Institute for Public Health and the Environment, Bilthoven, the Netherlands; 2 Institute for Parasitology and Tropical Veterinary Medicine, Freie Universität Berlin, Berlin, Germany; 3 Center for Experimental and Molecular Medicine, Academic Medical Center, Amsterdam, the Netherlands; 4 Laboratory of Entomology, Wageningen University and Research Centre, Wageningen, the Netherlands

**Keywords:** Lyme borreliosis, Tick-borne encephalitis, Anaplasmosis, *Ixodes ricinus*, Transmission cycles, Vaccines, Prevention

## Abstract

Lyme borreliosis (LB) and other *Ixodes ricinus-*borne diseases (TBDs) are diseases that emerge from interactions of humans and domestic animals with infected ticks in nature. Nature, environmental and health policies at (inter)national and local levels affect the risk, disease burden and costs of TBDs. Knowledge on ticks, their pathogens and the diseases they cause have been increasing, and resulted in the discovery of a diversity of control options, which often are not highly effective on their own. Control strategies involving concerted actions from human and animal health sectors as well as from nature managers have not been formulated, let alone implemented. Control of TBDs asks for a “health in all policies” approach, both at the (inter)national level, but also at local levels. For example, wildlife protection and creating urban green spaces are important for animal and human well-being, but may increase the risk of TBDs. In contrast, culling or fencing out deer decreases the risk for TBDs under specific conditions, but may have adverse effects on biodiversity or may be societally unacceptable. Therefore, in the end, nature and health workers together must carry out tailor-made control options for the control of TBDs for humans and animals, with minimal effects on the environment. In that regard, multidisciplinary approaches in environmental, but also medical settings are needed. To facilitate this, communication and collaboration between experts from different fields, which may include patient representatives, should be promoted.

## Background

*Ixodes ricinus* is a hard tick species that transmits pathogens of medical and veterinary importance. It has recently become clear that the bite of *I. ricinus* by itself can also cause meat allergy [1, 2]. *Ixodes ricinus*-borne infectious diseases are a considerable health concern in many European countries for several reasons.

First of all, the European Center for Disease Prevention and Control has predicted that the incidence of tick-borne diseases (TBDs) will rise in the near future [3]. Several studies describe a long-lasting increase in the incidences of the two most commonly reported TBDs, namely Lyme borreliosis (LB) and tick-borne encephalitis (TBE) in several European countries [4–9]. Another trend is that human infections and diseases involving other tick-borne pathogens (TBPs), such as *Anaplasma phagocytophilum*, *Borrelia miyamotoi*, *Neoehrlichia mikurensis*, spotted fever rickettsiae and *Babesia* species, are emerging or being (re)discovered. Indeed, the number of studies describing infections and disease cases involving these agents is accumulating in the literature [10–17]. The severity and incidence of TBDs, other than LB and TBE, is unknown, awareness is low and adequate diagnostic modalities are often lacking in routine settings. Many of these pathogens are also of veterinary relevance, not only for livestock, but also for pet animals [18–21].

Secondly, the reliability of the diagnosis of LB and the efficacy of antibiotic treatments are publicly being questioned, including by some self-proclaimed experts and medical doctors [22, 23]. The (inter)national guidelines on the clinical diagnosis with recommendations for supporting laboratory diagnosis and treatment appear to be a matter of continuous debate [24]. This has led to considerable societal unrest. Furthermore, as *I. ricinus* is often infected with multiple zoonotic agents, it is still unclear to what extent co-infections are able to affect the course of LB [25]. Doubts and uncertainties about the severity, symptoms, diagnosis and treatment of TBDs are widespread in the media, and give rise to uncertainties and controversies between patients and health providers. This rising concern for TBDs has contributed to the formation of LB interest groups in many European countries, who actively seek public and political awareness, particularly for LB. In the Netherlands, for example, an association for LB patients presented a petition with more than 70,000 signatures of concerned citizens to the parliament for more awareness and research on LB and political attention [26].

Together, these concerns require actions of public and medical health professionals and require solid, evidence-based solutions, to minimize the concerns and disease burden of TBD. This review aims to link the knowledge on *I. ricinus* and TBDs from different disciplines, in order to formulate possible solutions and knowledge gaps to control ticks and TBDs. An excellent review on the public health concerns and the challenges to control of *I. scapularis*-borne diseases in the USA has been published [27]. Despite the differences in ecology, epidemiology, environmental and health care systems, there are overlapping research questions which can be tackled together.

## Abundance and spread of *I. ricinus*

Understanding which factors drive population densities of disease vectors is an important step in assessing disease risk and formulating possible intervention strategies. *Ixodes ricinus* has a four stage life-cycle, i.e. egg, larva, nymph and adult, requiring only one blood meal during every active stage. The time for *I. ricinus* to complete its life-cycle varies between three and six years, mostly depending on climate and host availability. *Ixodes ricinus* employs an ambush strategy for host finding [28], which implies climbing the vegetation, clinging to the tips of stems, and waiting for a vertebrate host. Questing ticks cling to a host animal as the animal passes through vegetation. After feeding for a few days, ticks detach from the host and fall in the litter layer. It takes several months to molt into their next developmental stage, or, in the case of adult females, to lay several thousand eggs and subsequently die. Only a small fraction of the ticks complete the life-cycle: about 10% of the questing larvae will develop into a questing nymph, and then again between only 1 and 10% of the nymphs manage to develop into a questing adult.

Although *I. ricinus* can utilize a multitude of host species, these host species differ considerably in the numbers of ticks they feed, which further differs between the different tick life stages. In forest areas, larvae predominantly feed on rodents, nymphs feed on the highest variety of host, but mostly forest birds and rodents, whereas the key reproduction hosts for ticks are deer [29]. Although annual fluctuations in rodent densities affect the densities of nymphs the following year to some extent, the (local) presence of propagation hosts, mostly deer, is often the key factor for the presence of moderate tick densities in forested areas [30]. *Ixodes ricinus* spends almost its entire life in the vegetation. Temperature and relative humidity are key requirements for the development, survival and activity of *I. ricinus.* They are considered to be the principal factors limiting the geographic range of *I. ricinus* [31–33]. More locally, the survival time of ticks also strongly depends on (micro)climatic conditions. The large spatiotemporal fluctuations in the densities of questing ticks within a location is mostly determined by daily and seasonal weather conditions [34]. More generally, the climatic changes over the last decades have probably resulted in an increased length of the annual tick questing season [35], whether that has affected the population sizes of ticks is unknown.

These key requirements imply that *I. ricinus* is mainly found in deciduous woodland containing small mammals and deer, but in some areas with sufficient rainfall, large populations may occur in open habitats such as meadows, dune areas and moorland, where the majority probably feed on livestock [36]. Although very focal and often in low densities, *I. ricinus* has also been found in green areas in cities, such as parks and gardens [37, 38]. There, hedgehogs, rather than deer, might act as propagation hosts [39, 40].

## Policy driven changes in abundance and spread

Although direct evidence is lacking, the increase in LB and TBE incidence is very likely caused in part by the increase and spread of *I. ricinus* populations [41]. Tick-suitable areas in Europe are expanding, particularly due to reforestation and other actions to restore and protect nature [35, 42–47]. For example, the protective status of wildlife has resulted in increases in their abundance and spread, particularly of deer populations. Expanding and creating ecological networks across Europe is not only beneficial for wildlife, but also for ectoparasites and their associated pathogens, allowing easier maintenance and spread to new areas. The current policy of some European countries is to create more green spaces in (sub)urban areas to improve human health and well-being, and to mitigate the effects health risks such as heat wave, air pollution and flooding (Committee on Climate Change 2014). It is important to realize, however, that these spaces may also enhance opportunities for contact between humans and *I. ricinus*, posing risks for acquiring TBDs [38, 48–50].

### Transmission dynamics of TBDs

Pathogens can be acquired by ticks while feeding on infected hosts. In suitable tick vectors, TBPs have the ability to persist throughout the molting process to the next instar, a phenomenon called transstadial transmission. The efficiency of vertical transmission, from female tick to her offspring, varies from non-detectable for *B. burgdorferi* (*s.l.*) and *B. microti* [51, 52], to ~40% for *B. venatorum* [53] to close to 100% for *R. helvetica* [54]. *Ixodes ricinus* is capable to transmit more than twenty different (potentially) pathogenic parasites, bacteria and viruses via their blood meal to vertebrate hosts (Table 1). Pathogen transmission by ticks requires many often unexplored tick-pathogen interactions, from the migration of these pathogens from the gut to their secretion in tick saliva [55].

**Table 1:** Pathogens detected in, or transmitted by, *I. ricinus.* Pathogens are defined here as microorganisms which have been implicated in disease, because of evidence of infection in patients. Some pathogens have only caused disease in immune compromised cases. For most pathogens the Koch’s postulates have not been fulfilled and solid epidemiological evidence is lacking too [217]. Some pathogens, particularly *Bartonella, Francisella* and *Coxiella*, have other main modes of transmission. Transmission of *Hepatozoon* spp. by *I. ricinus* is not proven*,* but the infection of animals with *Hepatozoon* spp. usually involves the digestion of infected ticks. Finally, human infections of TBEV have also occurred through ingestion of contaminated, unpasteurized milk products [218], and other tick-borne pathogens have been transmitted *via* blood transfusion

Microorganism	Variants	Disease	Reference
*Borrelia burgdorferi* (*s.l.*)	*B. afzelii*	Human	[23, 91, 219]
	*B. garinii*	Human	
	*B. burgdorferi* (*s.s*.)	Human/ animal	
	*B. spielmanii*	Human	
	*B. valaisiana*	Human	
	*B. bavariensis*	Human	
	*B. bissetti*	Human	
	*B. finlandensis*	–	
	*B. lusitaniae*	Human	
	*B. turdi*	–	
*Babesia* species	*B. venatorum*	Human/animal	[220–222]
	*B. divergens*	Human/ animal	
	*B. microti*	Human	
	*B. capreoli*	Animal	
	*B. odocoilei*(*-*like)	–	
Spotted fever rickettsia	*R. helvetica*	Human	[223]
	*R. monacensis*	Human	
*Anaplasma phagocytophilum*	Ecotype I	Human /animal	[224]
	Ecotype II	–	
*Borrelia miyamotoi*	Russian	Human	[17]
	European	Human	
*Neoehrlichia mikurensis*		Human/animal	[225, 226]
*Spiroplasma ixodetes*		Human/animal	[227, 228]
Orbivirus	Kemerovo virus	Human	[229]
	Lipovnik virus	Human	
	Tribeč virus	Human	
Flaviviruses	Tick-borne encephalitis virus	Human	[18, 230, 231]
	Louping ill virus	Animal/ human	
Nairovirus	Grotenhout virus	–	[232]
Coltivirus	Eyach virus	Human	[230, 233]
Phlebovirus viruses	Uukuniemi(-like) virus	–	[234]
*Midichloria midichondria*		–	[235]
*Hepatozoon* species		–	[236]
*Coxiella burnetti*		Human/animal	[237]
*Francisella tularensis*	*F. tularensis holarctica*	Human/animal	[238, 239]
*Bartonella* species		Human	[240]

Vertebrate hosts can be regarded best as amplifying hosts for TBDs, their prominent role is to produce a sufficient number of newly infected ticks to close the enzootic cycles of pathogens. The infection dynamics of pathogens in vertebrate hosts varies in host range, tissue tropism and infection time. The host range of some pathogens, for example *B. lusitaniae* and *A. phagocytophilum* ecotype II, is relatively small with only a few vertebrate species being able to act as amplifying hosts, whereas the host range of others, such as *B. afzelii* and *A. phagocytophilum* ecotype I, is much broader. Tissue tropism varies from skin (*B. afzelii*), to blood (*Babesia* species), immune cells (*A. phagocytophilum*, *N. mikurensis*), endothelium (*R. helvetica*) and even to the central nervous system (TBEV, *B. garinii*). Sometimes, adequate immune responses are developed, for example against *B. miyamotoi* and TBEV, giving rise to short-term, limited infections. Other pathogens, such as *B. burgdorferi* (*s.l*.), *A. phagocytophilum* and probably also several *Babesia* species too can evade the immune system and cause chronic, long-lasting infections. Infections with *B. garinii* appear to be latent in thrushes (*Turdus iliacus*) for several months, but can then be reactivated by physiological cues [56]. Recurrent bacteremia also occurs in sheep, which remain infected persistently with *A. phagocytophilum* [57].

Transmission dynamics can also be affected at the (vertebrate) community levels *via* many, often poorly understood, mechanisms. For example, most vertebrates often are simultaneously or sequentially infected with multiple pathogens. Patterns of (co-)infection arise because infection by one microorganism affects susceptibility to others or due to inherent differences between hosts [58]. Another example is the dilution effect hypothesis, where diluting the abundance of transmission-competent hosts with non-competent hosts will reduce the probability of ticks feeding on transmission-competent hosts and consequently decreases the infection prevalence of pathogens in ticks [59]. This mechanism probably applies only in certain circumstances for a few TBPs, and even less often if considering abundance rather than prevalence of infected ticks [60]. Recently, we showed that mesocarnivores can lower the number of ticks feeding on reservoir-competent hosts, which implies that changes in predator abundance may have cascading effects on tick-borne disease risk [61].

Transmission dynamics can also be affected by weather and climatic conditions. The seasonal synchrony of larval and nymphal stages is an important driver of non-systemic transmission of TBEV *via* co-feeding of infected nymphs with uninfected larvae. This synchrony in tick activity and feeding, in turn, is affected by temperature patterns, in particular autumn cooling and spring warming [62, 63]. Climate change might therefore not only affect the distribution of ticks themselves, but also the distribution and nymphal infection rate of TBEV, and maybe also of other TBPs [41, 64].

These infection dynamics are important drivers for the abundance and spread of infected ticks, and therefore have major clinical implications, implications on the incidence, but also on the risk management and control of the associated diseases. For example, the geographical distribution of TBEV is multifocal [63] with relatively low infection rates, whereas Lyme spirochetes are more widespread with relatively high infection rates in *I. ricinus* [65]. Five genospecies of *B. burgdorferi* (*s.l*.) are commonly associated with LB in Europe: *B. afzelii*, *B. garinii*, *B. burgdorferi* (*sensu stricto*), *B. spielmanii* and *B. bavariensis* [23]. *Borrelia afzelii* is predominantly involved in cutaneous manifestations, such as erythema migrans (EM) and acrodermatitis chronica atrophicans (ACA), *B. garinii* and *B. bavariensis* in neuroborreliosis (LNB), and *B. burgdorferi* in Lyme arthritis (LA) [23, 66]. The incidence of the different manifestations of LB can be partially explained by their pathogenicity and by the relative occurrences of different genospecies in questing ticks [34]. Specific associations have also been found between vertebrates and *Borrelia* genospecies. For example, rodents and voles appear to contribute most to the transmission cycle of *B. afzelii*, whereas thrushes contribute most to the *B. garinii* and *B. valaisiana* cycles [29]. It is to be expected that local abundances of these animals in tick suitable recreational areas determine the risk of acquiring the specific disease manifestations [38, 45].

### Epidemiology of TBDs

Measuring incidences and cost of illness (humans) or production loss (livestock) can guide decision-makers to prioritize health policies and initiate cost-effective actions to control diseases with the highest economic or societal impact [67]. As TBE is notifiable in many European countries, incidences and sometimes also cost of illness have been estimated [68]. This information has enabled the calculation of the cost-effectiveness of vaccination strategies against TBE in several countries [69], which further aided the formulation of various strategies to control TBE, from creating awareness alone to incidence-, travel- or profession-based vaccination advises to mass-vaccination campaigns [9, 70].

The epidemiology of LB is more complex. In most European countries LB is not notifiable and incidence estimates are often based on passive reporting laboratory surveillance or on incidental, systematic investigations [71, 72]. Early stages of LB are underreported in laboratory surveillances, because most cases are serologically negative at presentation. Furthermore, laboratory testing at that stage is often not required, hence not recommended by guidelines, for the diagnosis of EM. Most importantly, the clinical manifestations of LB differ enormously in incidence and disease burden. In the Netherlands, 95% of the LB cases are EM, 2% LNB, 2% LA, 0.9% ACA, 0.4% borrelial lymphocytoma, 0.1% Lyme carditis and 0.1% had ocular manifestations [71]. In Germany, comparable proportions were observed [73]. A recent study estimated the total disease burden of LB for the Netherlands. Although ~91% of the LB cases had EM, it only constitutes ~6% of the disease burden, whereas the ~5% cases which displayed persisting symptoms attributed to LB accounted for almost 90% of the disease burden [74]. Thus, controlling the incidence of patients with persisting symptoms attributed to LB, will have the highest impact on reducing disease burden, but hardly on the disease incidence.

Only a few studies have investigated the incidence of other TBDs transmitted by *I. ricinus*, such as anaplasmosis and babesiosis, in Europe [75]. For example, a sero-epidemiological study estimated between 10 and 40 human anaplasmosis cases in Belgium per year [11, 76]. Human cases of other TBDs are being reported in the literature, mostly as case studies or series. In contrast, the exposure through bites of infected ticks in the general population and in risk groups such as forest workers is high. Based on molecular evidence alone, the probability of infection with a TBP other than Lyme spirochetes after a tick bite is roughly 2.4% [12]. Similarly, among patients with EM, the probability of a co-infection with another TBP is approximately 3% [12]. How often these infections cause disease or to what extent co-infections affect the course of LB needs further investigation. Infections with TBDs in humans is supported by many serological studies where antibody titers against for example *A. phagocytophilum* have been found in a few percent of human populations [77–79]. Nonetheless, the incidence and severity of the medical problems caused by these TBPs in many, if not all, European countries are unknown. One of the reasons for that is that current diagnostic tools for many of the TBDs are non-existing, of questionable quality, or poorly validated in the European setting. As a consequence, the awareness of other TBDs among physicians and the public is generally low. Therefore, to gain more knowledge on the incidence and nature of TBDs it is imperative to improve laboratory diagnostic tests and awareness.

Domestic animals are more prone to exposure to ticks than humans, as they generally spend more time outdoors, are in closer proximity to the ground and vegetation, and have coats that facilitate tick attachment. Since none of the TBDs associated with *I. ricinus* are notifiable in Europe, official information on TBD incidence in animals is not available. One exception is Q-fever caused by *Coxiella burnetii*, but the role of ticks in the epidemiology of the disease is considered to be negligible [80]. Most reports concern case descriptions, seroprevalence studies or molecular surveys looking at the occurrence of pathogens in ticks collected from the vegetation or animals, which says little about the actual incidence of clinical disease in animals.

## Clinical aspects of TBDs: clinical presentation, diagnostics and treatment

### Clinical presentation of LB and TBE

**Fig. 1:**
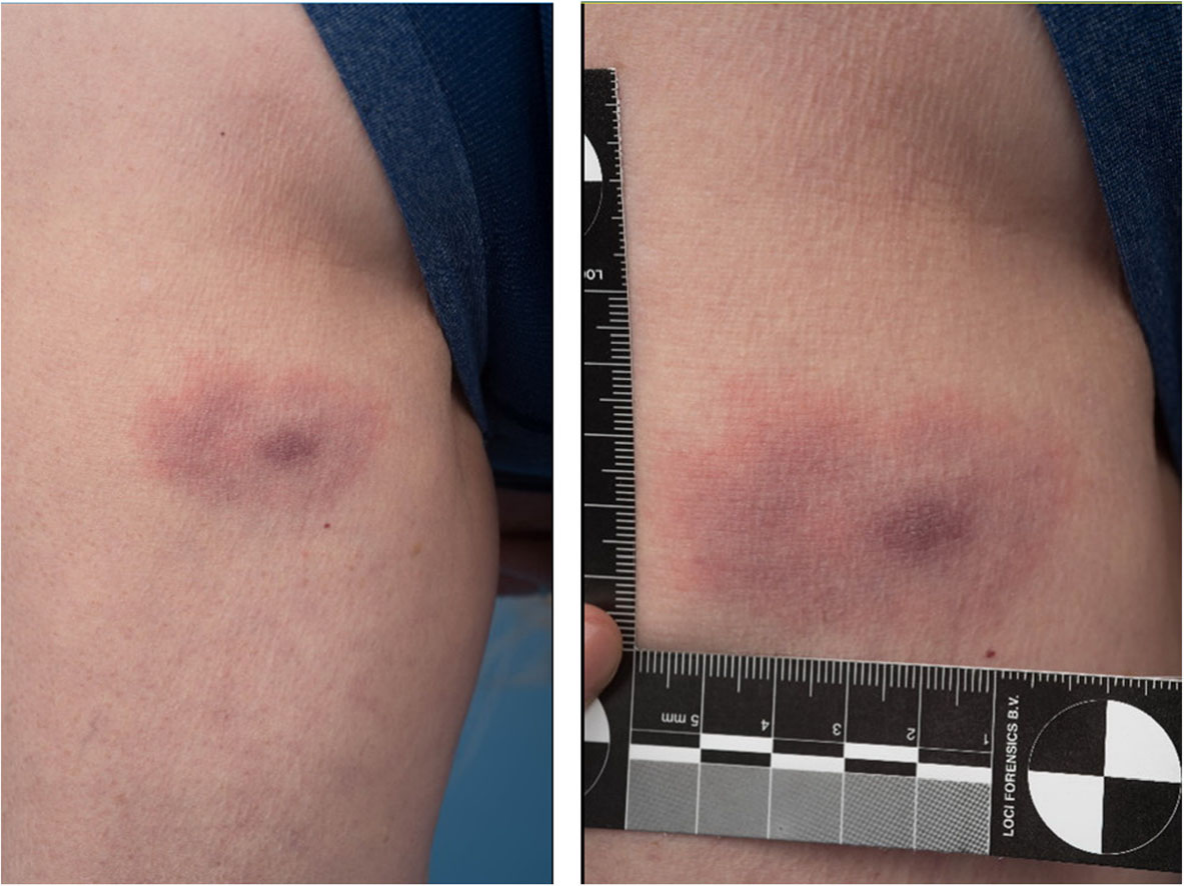
Clinical representation of an EM, the most common manifestation of LB. A culture-proven EM (diameter ~6 cm) on the leg of a 62-year-old female. This patient presented with this slowly expanding macula with very faint central clearing as the only symptom. There was no known tick bite prior to the development of the lesion.

LB is divided in three partially overlapping stages, reflecting the duration of the infection and the severity of the disease [23, 81]. The first stage is characterized by the hallmark EM, an erythematous expanding skin lesion at the site of the tick-bite (Fig. 1), usually occurring 1–2 weeks after the tick-bite. When left untreated or unnoticed the infection can disseminate and cause early disseminated and eventually late disseminated LB, the second and third stage respectively [81]. The characteristic manifestations of early disseminated LB include other skin manifestations, such as lymphocytoma and multiple EM, carditis, oligoarthritis and neurological symptoms, such as meningo-(poly)radiculitis (Bannwarth syndrome) with or without cranial nerve involvement [82], amongst other rare manifestations. The central nervous system or the joints can also be affected in late disseminated disease, but the hallmark clinical manifestation of late LB is ACA [83].

Apart from these clear-cut manifestations, there are patients with non-characteristic complaints such as myalgia, arthralgia, fatigue, which are sometimes attributed to LB. These complaints are often long-lasting and can even be debilitating. The constellation of these symptoms is sometimes referred to as chronic Lyme, however this term seems to be an umbrella name for a variety of diseases and syndromes [22, 84]. Examples thereof include late disseminated LB, post-treatment LB syndrome (a post-infectious syndrome), a persisting *B. burgdorferi* (*s.l*.) infection after antibiotic treatment, or one of many other diagnoses misattributed to LB [85]. How to define “chronic Lyme” is more than a semantic discussion as a proper diagnosis [84], i.e. the identification of the nature and cause of an illness, greatly determines the care and cure of patients [85], and could lower the disease burden and medical costs.

Exposure to Lyme spirochetes in animals in Europe is common, with reported seroprevalences in Europe in healthy dog populations ranging from 0.3% in southern Italy [86] to 26% in Serbia [87] and 7% in horses from in Italy [88] to 30% in France [89], but clinical disease with a conclusive LB diagnosis is rare. Clinical LB caused by *B. burgdorferi* (*s.s*.) has nonetheless been reported in dogs, horses and cats [90–95]. A broad spectrum of clinical signs has been associated to *Borrelia* infections in animals, including fever, lethargy, weight loss, (shifting) lameness, ataxia, uveitis, polyarthritis, glomerulopathy and neuritis [96–98]. This variation might to some extent be the result of unapparent co-infections with other pathogens, such as *A. phagocytophilum* [99, 100].

*Ixodes ricinus* ticks transmit the European variant of TBEV [101]. Although the majority of human infections are asymptomatic, the first symptoms are displayed between 2–28 days (median = 8) after a tick bite [102, 103]. Infections with the European TBEV usually display a typical biphasic course with a viremic phase of 2–10 days and a neurological phase of 1–21 days, separated by a period (median = 7 days) without symptoms [104]. In the first phase the most common symptoms are fever, fatigue and headaches [103]. After a temporary recovery, neurological symptoms appear in the second phase, ranging from mild meningitis to severe meningoencephalomyelitis [103]. Although TBEV-EU mortality is relatively low (1–2%), neurological sequelae, which can greatly affect the quality of life, often occur [105]. TBEV may also affect dogs and result in fever, change in behavior and various neurological symptoms (reviewed in [106]). A variant of the TBEV, Louping Ill virus causes acute encephalomyelitis, resulting in severe illness and death in livestock, especially sheep and red grouse [18].

### Clinical presentation of other TBDs

The clinical spectrum of human granulocytic anaplasmosis ranges from subclinical and self-limiting to subacute, chronic or severe in the immunocompromised [107]. The incubation period is 1–2 weeks, after which non-characteristic symptoms (e.g. fever, flu-like symptoms) arise, accompanied by nausea, vomitus, abdominal pain and arthralgia in approximately one-third of the patients. A skin rash or neurological symptoms are less frequently observed, and the estimated fatality rate is less than 1% [108–112]. Animal species that may be affected by *A. phagocytophilum* include dogs, cattle, horses and sheep and clinical signs vary in severity but are usually non-specific such as fever, lethargy and anorexia [113]. To date, there are dozens of case reports of human neoehrlichiosis, the disease caused by *N. mikurensis,* describing acute and chronic infections characterized by fever, headache, nausea, arthralgia, haemorrhages and weight loss [16]. *Borrelia miyamotoi* causes hard tick-borne relapsing fever (HTBRF). The onset of symptoms starts approximately two weeks after a tick-bite with a sudden onset of high fever with signs of septicaemia accompanied by headache, myalgia, arthralgia, and coughing or even gastrointestinal symptoms. The relapsing fever episodes typically last three days, divided by seven relatively healthy days, although for HTBRF the typical relapsing fever pattern is not that often observed [114]. The general trend of the course of the disease is subsiding and self-limiting, but in rare cases, i.e. in highly immunocompromised individuals, the disease appears to be neuro-invasive [114–118]. The two spotted fever rickettsias transmitted by *I. ricinus* are *R. helvetica* and *R*. *monacensis.* Their infections may cause vasculitis with fever, headache, myalgia and local lymphadenopathy. An inoculation eschar and generalized maculopapular rash, which are pathognomic for other spotted fever rickettsiosis, are rarely described for these genospecies [115, 119, 120]. The pathogenicity of *R. helvetica* is only partly established by several case series and reports in Europe [121–126]. *Rickettsia monacensis* infection is even less defined, although culture, molecular and serological evidence of human exposure has been reported [127–129].

In Europe, human babesiosis is caused by *B. microti, B. divergens* and *B. venatorum* [12, 130–132]. All *Babesia* species infect erythrocytes and cause haemolysis, leading to the clinical manifestations of fever, anaemia, jaundice, haemoglobinuria and potentially also renal insufficiency. Over 40 human babesiosis cases have been reported in Europe, mostly in asplenic patients. Other risk factors are immunosuppression, depletion of mature B-cells and old age [21]. Judging from the discrepancy between case reports and seroprevalence, an asymptomatic and/or self-limiting course is common [133]. Although serious infection appears to be uncommon, when acquired, the disease has a mortality rate of 42% in *B. divergens* and 5% in *B. microti* [134]. *Babesia divergens* is the causal agent of bovine babesiosis in Europe. The clinical picture is similar to that seen in humans, with a bimodal seasonal occurrence of the disease that is associated with *I. ricinus* activity. In cattle, an inverse age resistance phenomenon is present in which calves up to the age of 9–12 months are susceptible for infection, but resistant to disease [21].

Human infections with multiple TBDs and even with non-tick borne pathogens have been described [135, 136]. Co-infections have been shown to affect the course of LB causing a longer and more disabling course of disease [137–142]. Although co-infections in ticks are the rule rather than the exception [25, 34], the opposite is probably true in humans: co-infections seem to occur only occasionally [129, 143–146]. Our recent findings indicate that among patients with EM the probability of a co-infection with second TBP is merely 3% [12]. To date, there is no convincing evidence that infection with any other TBP or any other infectious agent, is associated with chronic Lyme [147, 148].

### Diagnosis of LB

In diagnosing LB, the foremost tool for a physician is a thorough history and physical examination. An EM is considered a clinical diagnosis and additional laboratory testing for EM is discouraged [82]. For many other disseminated forms of LB, laboratory work-up, including a search for alternative explanations, may serve to aid the physician. Serology is the current standard as it has good diagnostic parameters with a sensitivity and specificity of more than 90–95% in LB patients with late (disseminated) manifestations [149]. Serology has some disadvantages. First, the sensitivity is low in early stages of LB, approximately 50% [149], which may lead to a wrong or delayed diagnosis. Secondly, approximately 5% of the general population - and even higher depending on the age, geographical region and the population examined - have antibodies against Lyme spirochetes, while not having active LB [150]. Thus, serology cannot always differentiate well between a past and a current *Borrelia* infection [151–153]. Additional tests include PCR or culture, which are only recommended for specific manifestations and specific tissues or fluids: on synovial fluid/tissue in the case of LA, on a skin biopsy in the case of an ACA or in some specific cases of LNB on cerebrospinal fluid (CSF) [154, 155]. For LNB, other laboratory tests are available, such as leukocyte count, intrathecal antibody production, or intrathecal CXCL-13 concentration, to support the presence of an infection in the central nervous system or other inflammatory conditions [156].

The diagnosis of any form of chronic Lyme is far more complicated. Some of these patients may benefit from (additional) antibiotic treatment, while others may be better helped with other forms of treatment or rehabilitation. A laboratory test that is able to adequately differentiate between a past and active *Borrelia* infection is desired for these patients. It has been hypothesized that cellular tests have this ability. Several of these tests are already commercially available, but their accuracy has not been adequately determined [157–160] and, therefore, warrants more research before they can be used in clinical practice [159]. In addition, there are many alternative methods, which are said to test for LB, but sound evidence for these methods (e.g. as dark field microscopy directly on blood, VEGA-test or bio-resonance) is lacking [161].

When deciding to test for a given condition, whether it be LB or any other disease, it is important not to only take into consideration what the technical performance of a test is, but to also consider the pre-test probability that the patient has the disease [155]. When the pre-test probability of LB is low, then - taking into account the current diagnostic parameters of serological tests and the incidence of IgG-seropositivity in the general population - the added value of testing is limited. Furthermore, the various LB manifestations, the pre-test probabilities as well as the population under study and their expectations vary greatly between primary, secondary, and tertiary care. This might also affect recommendations for the use of *Borrelia* serology in current guidelines and requires further investigation. In that regard, although not recommended in most guidelines, it could be argued that testing for LB in patients with longer-lasting symptoms with a low pre-test probability in the primary care setting, could actually be helpful. In this situation, a negative test result would make an LB manifestation extremely unlikely, whereas a positive test result would require further investigation, e.g. referral to secondary or tertiary care center. Nevertheless, in some situations testing for LB is discouraged altogether, specifically when the patient is clinically diagnosed with an EM.

The non-specific clinical picture, together with a high seroprevalence, also complicate the diagnosis of LB in animals. The combination of a history to tick exposure within an endemic region, clinical signs consistent with LB, a positive test result, exclusion of differential diagnoses and response to treatment are required for a presumptive LB diagnosis in animals [162].

### Diagnosis of TBE and other TBDs

TBEV infection is associated with general non-specific infectious biochemical and blood count results. CSF analysis usually shows pleocytosis with polymorphonuclear cells early, and mononuclear cells late, in the disease development [163]. Serology can be performed on both liquor and serum by IgM/IgG ELISA, which is the most common diagnostic method for TBEV-infection in dogs as well [106]. A four-fold rise in TBEV-specific antibodies in liquor or serum confirm the diagnosis. A neutralization assay is recommended in flavivirus endemic regions to avoid a false positive result [104, 164]. Imaging of the brain and/or myelum may result in focal abnormalities; however it does not contribute greatly to the diagnosis [163].

Diagnosis of other TBDs is based on the assembly of specific clinical characteristics, laboratory findings together with diagnostic tools in a setting of relevant epidemiological exposure. The main non-specific laboratory findings associated with other TBDs are general parameters found in infection, such as elevated inflammation parameters (C-reactive protein, erythrocyte sedimentation rate), leukopenia or leucocytosis, thrombocytopenia and anaemia, with or without elevated liver enzymes or kidney dysfunction. Especially in *A. phagocytophilum* and *N. mikurensis* infection, leukopenia is observed due to leukocyte infection [108, 165]. *Babesia* spp. can cause a distinct haemolytic anaemia due to erythrocyte infection with accompanying elevated bilirubin, reticulocytosis and decreased haptoglobin [166]. Thrombocytopenia appears to be most pronounced in anaplasmosis, babesiosis and HTBRF. In the rare severe cases of anaplasmosis and HTBRF with involvement of the central nervous system, the CSF can reveal pleocytosis [167, 168]. For some TBDs, there are additional, more specific, tests available, such as a buffy coat examination for *A. phagocytophilum* or peripheral blood smear with Giemsa staining in *A. phagocytophilum* and *Babesia* spp. to look for respectively morulae or merozoites by microscopy [169, 170].

Most additional targeted diagnostic tests in TBDs are either in the experimental phase or not widely validated (molecular tests), based on cross-reactivity between other species (serology), time-consuming, or difficult to perform and requiring a high level of expertise (cultures) [171–173]. In general, the sensitivity of available molecular tests for all these TBDs is high in the first week of disease and rapidly decreases over time, and after proper treatment. Therefore, a positive PCR result is helpful, but a negative result does not rule out the diagnosis. As an exception to the rule, *Babesia* spp. can be detected up to months to years after (un)treated infection [174, 175].

For most TBPs, there are no standardized antigens, or well-defined consensus as to what thresholds constitute a significant antibody titer. As a rule of thumb, serological tests are usually required to show a four-fold rise in antibody titer in convalescent sera. It should be noted that the onset of symptoms sometimes precedes the rise in antibody titer. In addition, because antibodies may persist beyond the clearance of infection, it can be difficult to distinguish between a past, recent or current infection [21, 169, 176]. For *A. phagocytophilum*, *Babesia* and *Rickettsia* spp. indirect fluorescent antibody tests are available, yet they make use of other strains or even genospecies than the ones found in Europe, with the exception of *B. microti* [166, 171, 177, 178]. Serological tests for *B. miyamotoi* are in the experimental phase and based on specific antigens (glycerophosphodiester phosphodiesterase (GlpQ) and more recently also variable major proteins (Vmps) identified in the available different isolates from Asia and the USA [179, 180]. These assays do not discriminate between the different relapsing fever *Borrelia* genospecies. There is no widely available and established serological test for the diagnosis of *N. mikurensis* infection*.*

### Treatment of TBDs

LB is treated with antibiotics. The prognosis, especially when treated early in the course of the disease is good, although rarely antibiotic failure can occur. In contrast, persisting symptoms can be observed in approximately 5–20% of LB patients despite recommended antibiotic treatment [181, 182]. Therefore, this condition has been referred to as post-treatment LB [169]. It has been shown in multiple placebo controlled randomized trials that prolonged antibiotic treatment is not effective in treating these non-specific yet disabling and long-lasting symptoms [183–187]. LB in animals is also treated with antibiotics, usually with doxycycline given *per os* at 10 mg/kg every 12 or 24 h for a period of one month [162, 188]. In horses, the intravenous administration of oxytetracycline (5 mg/kg/day) was more effective in clearing experimentally induced *Borrelia* infections than doxycycline treatment [189].

There is no causal treatment for TBEV. Treatment consists of supportive care and there is no evidence that steroids or immunoglobulins are beneficial [104]. Asymptomatic or subclinical infection frequently occurs for all of the other TBDs and thus infection does not necessarily require treatment. However, when symptomatic, treatment is, or may be, warranted. The large group of intracellular other TBDs, such as *A. phagocytophilum*, *N. mikurensis* and spotted fever rickettsia, as well as *B. miyamotoi* are all susceptible to doxycycline, which is the drug of choice [116, 169, 173, 190] for adults. For younger children, pregnant women and when the central nervous system is affected, specific alternatives exist. Therefore, in countries where doxycycline is recommended as the first line treatment for LB, these pathogens would be concomitantly treated. In countries where beta-lactams are the drug of first choice for LB, clinicians should have a higher level of suspicion for other TBDs, since these are likely not co-treated as such. Moreover, babesiosis requires a different treatment, consisting of azitromycine and atovaquone or clindamycine and quinine depending on the severity of the disease [169]. Cattle suffering from babesiosis are treated with imidocarb diproprionate. In Africa, diminazene aceturate is frequently used to treat bovine babesiosis caused by *B. bovis* or *B. bigemina*, but this product is not available in Europe [21].

## Control of LB and other *I. ricinus*-borne diseases

Knowledge on *I. ricinus,* its associated pathogens and the diseases they cause have been increasing in many fields and many approaches to control or prevent TBDs have been investigated and proposed (Table 2). Excellent reviews and even (hand)books on this topic are available [191–195].

**Table 2:** Present and potential measures^a^ to control TBDs. This table is modified from Eisen & Gray [241]. There is not a single method that effectively controls all TBDs. National and local strategies, which combine several methods probably work best [191, 192]. Anti-tick vaccines blocking pathogen transmission in humans and domestic animals might encompass the silver bullet to control TBDs. Hygiene measures^b^ involve checking for tick bites, prompt removal, and most importantly, seek medical advice when developing symptoms (e.g. fever, skin rash) or illness in weeks to months after a tick bite

Personal	Domestic animal	Residential	Vegetation	Fauna	Medical
Avoid tick habitats	Avoid tick habitats	Xeriscaping/ Hardscaping	Awareness for visitors		Increase awareness and knowledge of medical doctors
Protective clothing	Treatments with topical or systematic acaricides	Keep grass short, remove weeds, remove leaf litter and brush	Reduce tick abundance on sites with high recreational activities	Deer fencing	Technical improvement of laboratory tests
Repellents	Hygiene measures^b^	Remove harborages/food for rodents and insectivores	Avoidance tick habitats/ directing visitor flows	Deer removal	Improvement of diagnostic/clinical pathways
Acaricide-impregnated clothing		Fencing to exclude wildlife	Mowing/extensive grazing of paths and recreational sites	Topical acaricide for propagation hosts (deer)	Improve cure and care of patients with late LB and persisting complaints
Hygiene measures^b^		Move play/rest structures to low risk areas	Create open habitats rather than woodlands	Sheep mopping	Prophylactic antibiotic treatment after a tick bite
Control ticks on dogs/ cats and in gardens		Chemical/fungal acaricides		Topical acaricide/antibiotics for rodents	
sTBE vaccine	TBE vaccine			Oral LB vaccine for rodents^a^	
LB vaccine^a^	LB vaccine			Oral tick growth regulator/acaricide^a^	
Tick vaccine^a^	Tick vaccine^a^			Tick vaccine^a^	

### Personal preventive actions

Control of *I. ricinus*-borne diseases primarily consist of the promotion of personal preventive actions for the public and for risk groups, such as forest workers, by providing information and education. Such actions include avoiding high-risk habitats, wearing protective clothing, application of repellents, prompt removal of attached ticks, and seeking medical advice when developing symptoms (e.g. fever, skin rash) or another illness in weeks to months after a tick bite. Personal protective measures have poor rates of compliance and their effectiveness has been difficult to demonstrate in terms of reducing disease cases [196, 197]. For example, providing information and education has not resulted in a decline in the incidence of LB in the Netherlands, not even after intensified efforts since 2003 [7].

### Environmental-based approaches

Environmental-based approaches mostly rely on reduction of tick suitable habitats, the disruption of the tick life-cycle or interference with pathogen transmission. A major advantage of environmental-based control options is that most of them can readily be applied in various practical situations, as they involve existing nature management options, such as mowing, grazing or fencing [195]. Furthermore, controlling tick abundance or tick exposure reduces the risk of acquiring any TBD for both humans and domestic animals. So far, there has been little interest in Europe in environmentally-based preventive measures. Large-scale and long-term spraying with acaricides was carried out in Russia during the 1970s and 1980s in an attempt to control *I. persulcatus*, the main vector of the TBEV [198]. The widespread application of acaricides has been publicly criticized and has become socially undesirable, because of their detrimental effects on the ecosystem and biodiversity [191, 199]. Unlike in the USA, only a limited number of studies exploring environmental-based methods to control ticks have been conducted in Europe [30, 195, 200–202]. A wide range of acaricidal products in various formulations, which are effective against *I. ricinus,* is being used for tick control on domestic animals [203].

### Health in All Policies

Most, if not all, of the available environmentally-based preventive and control measures suffer from the fact that they are not highly effective on their own [192]. Probably, long-term implementation of control strategies, i.e. the integrated use of two or more control measures, are necessary to effectively reduce disease risk. Only a few studies on the effectiveness of control strategies have been carried out in the USA, but not in Europe [192]. The successful implementation of environmentally-based preventive and control measures requires involvement of stakeholders from both nature management and human (and animal) health (‘One Health’). Of key importance is that the environmental control options for TBDs are put into the context of other aims and ambitions, such as nature conservation, ecosystem services or heat mitigation in urban areas. Indeed, sectors involved in nature management and environmental planning are often more familiar with a so-called ‘Health in All Policies’ approach. The ‘Health in All Policies’ approach integrates and articulates many health considerations, far broader than infectious diseases alone, into policymaking across sectors. A future challenge is to integrate the risk of TBDs, but also of wildlife- and other vector-borne diseases, into the ‘Health in All Policies’ in local nature organizations, such as Municipal Health Services and nature owners, but also governmental institutions and (inter)national organizations responsible for nature and health.

### Healthcare actions

More and better awareness of the epidemiology, clinical presentation and course of the various TBDs amongst physicians could raise a suspicion on these diseases in endemic regions. For example, the communication with health professionals on the presence of *B. miyamotoi* and TBEV in questing ticks in the Netherlands has resulted in the identification of the first cases of HTBRF and TBE [70, 168]. Clearly, there is room for the improvement of laboratory tests for the diagnosis of LB and especially other TBDs. Both direct (antigen tests, cultivation or molecular tests) and indirect tests (serology or cellular tests) could greatly aid in establishing the diagnosis. Rather than making one guideline for each tick-borne disease separately, it might be more advantageous to have one guideline for all TBDs for primary care centers with clear consensus on diagnostic testing and referral to secondary and tertiary care centers. Specialized guidelines for secondary and tertiary care centers can aid the diagnosis and treatment for more severe manifestations of LB, but also and for all variants of chronic Lyme. Finally, better knowledge on the course of the various diseases after treatment could prevent overdiagnosis and retreatment.

### Vaccination

Where the risk of infection is high or the resulting disease severe, vaccines may be the most efficient and cost-effective means of prevention and control [204]. TBE is well under control in Austria because of mass vaccination programs. The available TBE vaccines have an effectiveness of ~98%. With a vaccination rate in the population of 85%, it is estimated that more than 4000 severe cases of TBE were prevented in Austria between 2000 and 2011 [205]. Remarkably, the vaccination coverage in many central and eastern European countries is low [206], despite predictions that TBE vaccination programs in central and eastern Europe can be cost-effective [207]. A vaccine protecting against LB is currently unavailable in Europe, but a potential vaccine has recently been tested in a Phase I/II trial [208, 209], and another LB vaccine is being developed for the European market as well [210]. Based on the experiences with a previous Lyme vaccine that was on the American market, with an effectivity between 62% and 85%, it remains to be seen whether a Lyme vaccine will be widely accepted and used, or only cost-effective for high risk groups [211, 212]. Ideally, one would like to have a single vaccine for humans, protecting against multiple TBDs [213]. Anti-tick vaccines targeting other tick species already exist and are being used in the veterinary field. The strategy behind these vaccines is to locally control *Rhipicephalus* (*Boophilus*) tick species, and act as a safe and environmentally friendly alternative to acaricides [214, 215]. Application of anti-tick vaccines was shown to dramatically decrease the incidence of bovine babesiosis [216]. Whether anti-tick vaccines can also be used to (locally) eradicate *I. ricinus* populations and prevent human TBDs is difficult to predict due to its very large host range, yet is a topic of investigation [213].

## Conclusion

Unfortunately, there is no silver bullet to control TBDs yet. In order to effectively control TBDs, “health” should be considered in a broader context, involving ecosystems, the environment, wildlife, animals and also curative and public health and policymaking. This implies a multidisciplinary approach and asks for international collaborations throughout Europe, but also multidisciplinary collaborations and approaches at local levels. Patient representatives or patient advocacy groups are part of such a multidisciplinary approach. In our experience, patients and researchers often have shared goals and convictions, yet comprehensive collaboration in the field of LB research seems rare. Patient advocates can have a valuable role in anything from designing the study and securing funding, to effectively communicating study results to patients and the general public.

Since the ecology and epidemiology of TBDs are diverse, yet greatly influence the burden of the different TBDs, these should also be considered. In addition, more awareness amongst physicians, prompt recognition of the various clinical symptoms and improved diagnostic tools could aid in combating TBDs in the future. A variety of personal and environment-based preventive and control measures exist, but suffer from the fact that they are not highly effective on their own. Combining them, and investing in fundamental as well as translational research, to be able to formulate (evidence-based) strategies on the control of TBDs might prove to be the way forward. Last, but most certainly not least, for most of the TBDs no vaccine exists and therefore research should most definitely focus on vaccine discovery and development. In that regard, vaccines targeting the tick vector, which could potentially prevent multiple TBPs, have the potential to become the next silver bullet, and require further investigation.

## Abbreviations

ACA: Acrodermatitis chronica atrophicans
CSF: Cerebrospinal fluid
EM: Erythema migrans
GlpQ: Glycerophosphodiester phosphodiesterase
HTBRF: Hard tick-borne relapsing fever
LA: Lyme arthritis
LB: Lyme borreliosis
LNB: Lyme neuroborreliosis
TBD: Tick-borne disease
TBE(V): Tick-borne encephalitis (virus)
TBP: Tick-borne pathogen
Vmps: Variable major proteins
